# Mr. Leese, on Vaccine Inoculation

**Published:** 1800-10

**Authors:** L. Leese

**Affiliations:** Throgmorton Street


### *To the Editors of the Medical and Phyſical Journal.*

Gentlemen,

THE teſtimony of many reſpectable practitioners, and my own experience, having convinced me that the Cow-pock diſeaſe is leſs violent and leſs dangerous than the Small-pock in its mildeſt form; alſo that it is a ſure preventive of the Small-pock, if reſorted to previous to Small-pock infection; and if my opinion is correct reſpecting the period when Small-pock becomes infectious by the atmoſphere, I ſhall be able to prove that it will *prevent*, although the perſon may have received that infection two or three days. It is my preſent opinion that Small-pock becomes infectious many hours before the eruption is manifeſt on the ſkin. However this may really be, is not eaſily determined; and I know much diverſity of opinion exiſts on the ſubject. But the principal object of this letter is to offer for your inſertion ſome facts reſpecting the Cow-pock, which occurred in one family under my own obſervation, believing that ſome of the attending circumſtances were rather peculiar; and although the general opinion of the moſt diſcerning of the profeſſion, as well as of the public, now preponderates in favour of the new diſeaſe, yet as objections and prejudices are ſtill oppoſed to its progreſs, I truſt I ſhall not be accuſed cused of supererogation if I endeavour to throw my mite into the scale.

Master Teschemaker, 10 years of age, recently arrived from the West Indies, was attacked with fever on the 2d of July last; on the 4th, a variolous eruption appeared on the skin, with every appearance of a violent disease taking place. There were at this time four other persons in the house, relatives and attendants, who had not had the disease. I advised that they should immediately be inoculated with Cow-pock matter. They had not heard of the new disease, and of course did not at once adopt what I recommended; however, on the 6th, two of them, Mr. and Mrs. Krebs, adopted what I advised; and on the 7th, one other, a Negro woman. The fourth said he had Small-pock in the West Indies. On the 9th, Mr. K's arm manifested the impression of the disease; on the 12th, I found the pulse accelerated about 10, and his tongue a little affected. These symptoms continued about three days; the arm soon after scaled, and he became perfectly well. Mrs. K.'s arm did not show the same marks of disease until the 11th. On the 15th, it became very much inflamed, and fever occurred in about the same degree as in Mr. K. but she had much more head-ach, which I attributed in some degree to extreme anxiety and watchfulness, she having from Master Teschemaker's first attack attended him day and night unremittingly, and he was now in great danger. On the 16th, a pustule appeared on her leg, but the peculiar form and character I cannot describe, it having been broken very early by some external violence; I imagine by rubbing or striking the other leg against it.

This pustule on the leg seems to confirm Dr. Woodville's opinion, that such may be produced by persons inspiring a variolated atmosphere while under the other disease. The inoculated part as well as the pustule continued inflamed for about ten days, and this lady became well.

I was in doubt for several days, whether the one inoculated last (namely, a day after this lady and gentleman) had received the infection; but on the 17th, ten days after the inoculation, (she continuing to breathe an atmosphere highly variolated) some inflammation took place on the arm, and at the same time a considerable degree of fever; but there were many reasons to induce me to think this fever was the effect of cold, and nightly attendance on the miserable sufferer with Small-pock. From this she gradually amended for some days; but on the 26th, nine days after the first attack of fever, and nineteen after the Vaccine Inoculation, and after three days of renewed attack of fever, accompanied with violent head-ach and back-ach, a variolous and very numerous eruption appeared: The fever now abated, abated, and the inoculated arm ftill retained the appearance of local infection. It was now determined to fend her to the Small-pock Hofpital, where fhe recovered.

Mafter T——, the firft fubject mentioned with Small-pock died on the 27th. On the 31ft, Mafter Swain, about 10 years of age, another of the four, (who faid he had had the difeafe) began to ficken, and had a very violent and dangerous difeafe, but from which he is now recovered. Another young gentleman who had his paffage in the fame veffel, and who, moft probably, caught his difeafe from the fame fource as my firft, alfo died of Small-pock, within a few days of him; but this laft was not under my own obfervation, having taken lodgings at a different part of the town.

Now, Sirs, I muft take the liberty here to endeavour to imprefs on the Readers of your valuable Journal, (by a fort of recapitulation) the great utility of the new difeafe, for which the national gratitude ought, in fome way or other, to be prefented to Dr. Jenner. Here were fix perfons, all of them juft arrived from the Weft Indies, two of whom died of Small-pock, two others recovered only by a very fevere ftruggle and lafting deformity of the fkin; while the two who were inoculated (in time) with the Vaccine matter, efcaped by the means of a very flight difeafe, although they were for three days previous to their inoculation, and for fome weeks after, in the houfe and in the chamber with thofe who had Small-pock violently.

That the Negro woman was inoculated with Cow-pock matter, and yet had Small-pock, is not to be imputed to any want of power in that to prevent, but from its having been deferred too long; and at the time of inoculation I expreffed my doubt of its fuccefs.

If you think this detail worthy a place in your next Number, I fhall hope it will furnifh fome additional proof of the utility of the new Inoculation; and alfo, that fome of your Readers, of more experience and more difcernment than myfelf, may make fome deductions from thefe facts, to the further elucidation of this moft important difcovery. I am,

Gentlemen,

Your's, moft refpectfully,

L. LEESE.

